# Synthesis and characterization of core-shell Fe_3_O_4_-gold-chitosan nanostructure

**DOI:** 10.1186/1477-3155-10-3

**Published:** 2012-01-05

**Authors:** Hossein Salehizadeh, Elham Hekmatian, Meisam Sadeghi, Kevin Kennedy

**Affiliations:** 1 Department of Civil Engineering, University of Ottawa, Ottawa, ON, K1N 6N5, Canada; 2 Biotechnology Engineering Group, University of Isfahan, Isfahan, Iran; 3 Faculty of Medicine, Isfahan University of Medical Science, Isfahan, Iran

**Keywords:** bioseparation, core-shell, Fe_3_O_4_-gold-chitosan, hydrogel, magnetic, nanocomposite, nanoparticle

## Abstract

**Background:**

Fe_3_O_4_-gold-chitosan core-shell nanostructure can be used in biotechnological and biomedical applications such as magnetic bioseparation, water and wastewater treatment, biodetection and bioimaging, drug delivery, and cancer treatment.

**Results:**

Magnetite nanoparticles with an average size of 9.8 nm in diameter were synthesized using the chemical co-precipitation method. A gold-coated Fe_3_O_4 _monotonous core-shell nanostructure was produced with an average size of 15 nm in diameter by glucose reduction of Au^3+ ^which is then stabilized with a chitosan cross linked by formaldehyde. The results of analyses with X-ray diffraction (XRD), Fourier Transformed Infrared Spectroscopy (FTIR), Transmission Electron Microscopy (TEM), and Atomic Force Microscopy (AFM) indicated that the nanoparticles were regularly shaped, and agglomerate-free, with a narrow size distribution.

**Conclusions:**

A rapid, mild method for synthesizing Fe_3_O_4_-gold nanoparticles using chitosan was investigated. A magnetic core-shell-chitosan nanocomposite, including both the supermagnetic properties of iron oxide and the optical characteristics of colloidal gold nanoparticles, was synthesized.

## Background

Nanoparticles are nanostructures with at least one dimension being less than 100 nm. Gold-coated magnetic nanoparticles are a class of nanoparticles that have attracted much attention because of their advantageous characteristics, such as their inertness, non-toxicity, super magneticity, ease of detection in the human body, a magnetic core that is protected against oxidation, their facilitated bio-conjugating ability, catalytic surface, and their potential for a variety of biological applications [1,2]. Gold-coated nanoparticles have great biocompatibility with the human body with the ability to interact with biomolecules such as polypeptides, DNA, and polysaccharides [3]. Chitosan, poly-β-(1-4)-2-amino-2-deoxy-D-glucose, also has many favorable characteristics including: low toxicity and high biocompatibility. It has been widely used in many fields, such as water and wastewater treatment [4], biomedical applications as a drug carrier [5], therapy for repairing spinal damage [6] and for preserving nervous cell and mitochondrial membranes from harmful reactive oxygen species (ROS) [7]. The production of core-shell Fe_3_O_4_-gold-biopolymer nanocomposites has attracted much attention over the past several years as they can be used in biotechnological and biomedical areas, including biotargeting for cancer treatment, drug delivery, biodetection, and downstream processing (i.e., the purification and bioseparation of biomolecules). Gold nanocomposites utilizing chitosan offer several potential benefits using the magnetic core for controllability, as well as the immobilization of biomolecules and other optical properties through their gold shell [8-10].

This paper describes a simple and rapid method for synthesizing controllable, agglomerate-free Fe_3_O_4_-gold-chitosan nanocomposites. Glucose was used as the reducing agent and chitosan as the protecting and stabilizing agent. Additionally, the spectral properties of core-shell Fe_3_O_4_-gold nanoparticles synthesized by this method have been evaluated by modern analytical techniques and the results discussed.

## Materials and methods

### Synthesis of Fe_3_O_4 _nanoparticles

Fe_3_O_4 _nanoparticles were synthesized according to Ahmed et al. [11] with several modifications resulting in substantial quality improvements. All of the chemicals used in this research were of analytical grade and obtained from commercial sources. FeCl_2_. 4H_2_O, FeCl_3_·6H_2_O, sodium hydroxide, sulphuric acid, nitric acid, hydrochloric acid, *N*-tetra methyl ammonia hydroxide, formaldehyde (37%), ammonium hydroxide, sodium phosphate monobasic, sodium phosphate dibasic and hydrogen tetrachloroaurate(III) (HAuCl_4_.4H2O, 99%) were obtained from Merck, Germany. Chitosan was prepared from Sigma-Aldrich, USA. Deionized water was obtained from Milli Q system and used throughout. The solutions of FeCl_3_·6H_2_O (4 ml, 2 M) and FeCl_2_·4H_2_O (2 ml, 2 M) were prepared in 250 ml flasks, added to a flat bottom beaker, and stirred at 30°C for 45 min. The Fe(III)/Fe(II) ratio was kept 2 throughout. Then, an aqueous ammonia solution (100 ml, 1 M) was added by droplet under the cover of N_2 _gas and the pH of the solution was carefully adjusted up to 10. The solution was stirred for about 1 h until stable, black Fe_3_O_4 _particles appeared. Next, the particles were filtered and then rinsed with distilled water and then methanol until the pH reached 7. They were then dried in a vacuum oven at room temperature for 24 h.

### Synthesis of Fe_3_O_4_-gold nanoparticles

The synthesis of Fe_3_O_4_-gold nanoparticles was carried out according to Cui et al. [12] with some modifications. First, Fe_3_O_4 _nanoparticles were dispersed in a 0.1 M HAuCl_4_·4H_2_O solution in a flat bottom beaker for 20 minutes using sonication, and then slowly mixed in a shaking incubator at 38°C to allow the adsorption of Au^3+ ^into the Fe_3_O_4 _surface. Glucose was then added to the system as a reducing agent and the mixture was incubated at room temperature in a shaking incubator (200 rpm). The core-shell nanoparticles that formed were then washed with pure water until the pH reached 7.

### Synthesis of Fe_3_O_4_-gold-chitosan

Chitosan (200 mg) was added to 14 ml of acetic acid (1%, v/v) solution and stirred for 10 minutes at room temperature until it became a homogeneous viscous solution. Then, various concentrations of formaldehyde (2-10 ml, 5 M) were used to improve the gelation properties of the formed hydrogel. The prepared chitosan solution was simultanously added to the gold-coated magnetic nanoparticles being formed in the solution and incubated at room temperare with shaking in a shaking incubator (200 rpm) for 1.5 h leading to synthesis of the core-shell structure of Fe_3_O_4_-gold-Chitosan.

### Characterization

Fourier transformed infrared (FTIR) spectroscopy was carried out by a Bruker FTIR-6000 (Bruker, Germany) using KBr discs to investigate the interaction of functional groups in chitosan with the nanoparticles surface. The crystallographic characterization of nanoparticles was done by a powder X-ray diffraction (XRD) spectrometer (Bruker D8 Advance, Germany). Transmission electron microscopy (TEM) images to obtain the morphology and size of the nanoparticles were taken using a LEO920 TEM (Carl Zeiss, Germany). The topographic images of nanoparticles and their orientation in the chitosan texture were obtained by atomic force microscopy (AFM) (CSM-Bruker, Germany). The mean hydrodynamic diameter of nanoparticles was measured using Zetasizer (Malvern model, China),

## Results and Discussion

### Physical characteristics of Fe_3_O_4 _nanoparticles

**Figure 1:**
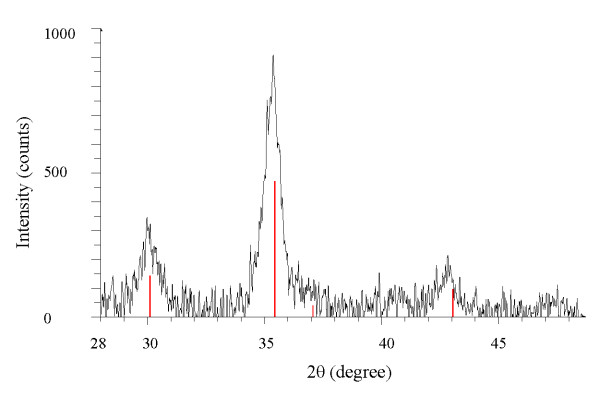
**XRD pattern of Fe_3_O_4 _nanoparticles with 9.8 nm diameter**.

**Figure 2:**
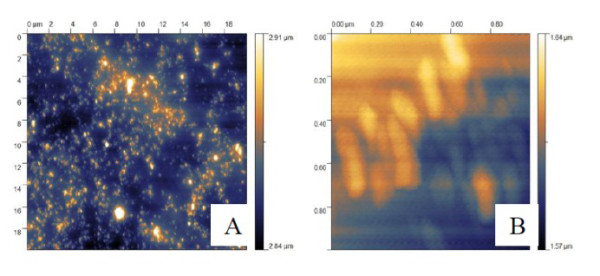
**AFM topographic images of physically dispersed Fe_3_O_4 _nanoparticles on chitosan gel (A), and immobilization of Fe_3_O_4 _nanoparticles in chitosan gel (B)**.

Gold magnetite nanoparticles have an Fe_3_O_4 _core with an average size of 9.8 nm in diameter. The average size of Fe_3_O_4 _nanoparticles was measured using XRD with Cu *Kα *radiation at 1.540 Å (Figure 1). Magnetic seeds were synthesized using co-precipitation under controlled condition (pH = 10) and N_2 _protection gas. The optimum mole Fe^3+^: Fe^2+ ^ratio used was 2:1. The AFM image of Fe_3_O_4 _stabilized by chitosan is exhibited in Figure 2. AFM topographic images indicate physically dispersed Fe_3_O_4 _nanoparticles on chitosan gel (A), and immobilization of Fe_3_O_4 _nanoparticles in chitosan gel (B). The magnetite nanoparticles were oriented in one direction due to magnetic properties (Figure 2-B).

**Figure 3:**
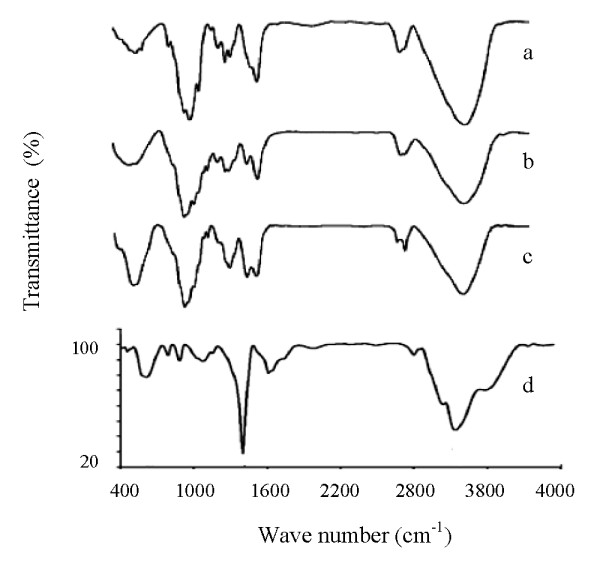
**FTIR spectra of a) chitosan, b) crosslinked chitosan by aldehyde, c) chitosan-Fe_3_O_4 _hydrogel nanocomposite (nanoparticles immobilized), d) Fe_3_O_4 _nanoparticles only dispersed on the surface of hydrogel (nanoparticles not immobilized)**.

The FTIR spectra of chitosan, formaldehyde cross linked chitosan hydrogel and Fe_3_O_4-_chitosan hydrogel are shown in Figure 3. The broad band found at 3429 cm^-1 ^is due to overlapped -OH and -NH groups in chitosan. The band observed at 2902 cm^-1 ^is attributed to C-H bands. The band at approximately 1656 cm^-1 ^is due to amide band C-O stretching, along with N-H deformation, and at 1592 cm^-1^, it is due to the characteristic peak of the NH_2 _group. The absorption peaks at 1412 cm^-1 ^are characteristic of -CH_2_- and, skeletal vibration involving C-O-C bridge stretching of the glucosamine residue is responsible for the band at 1107 cm^-1^. The 1025 cm^-1 ^band is likely related to CH-OH bonds in cyclic compounds. The peaks that appeared at 587 and 477 cm^-1^, are indicative of stretching, and the variation modes of Fe-O confirms the presence of crystalline Fe_3_O_4 _(Figure 3-c). For the Fe_3_O_4 _nanoparticles dispersed on the chitosan hydrogel, the FTIR spectrum confirmed considerable changes for the immobilized Fe_3_O_4 _nanoparticles based on the shape and frequencies of the bands, indicating the interaction of functional groups in chitosan with the Fe_3_O_4 _at the surface (Figure 3-d).

### Physical characteristics of Fe_3_O_4_-gold nanoparticles

**Figure 4:**
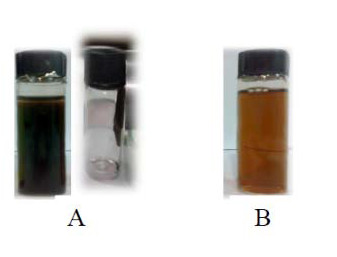
**Separation of Fe_3_O_4 _nanoparticles by magnetic effect (A), Fe_3_O_4_-gold core-shell solution (B)**.

Gold provides stability for the magnetic nanoparticles in solution as well as providing a good inert surface for assisting the binding of various biomolecules [13-15]. The gold shell was synthesized by the reduction of Au^3+ ^with glucose as a nontoxic, biocompatible reducing agent in the presence of Fe_3_O_4 _nanoparticles. When the Fe_3_O_4 _nanoparticles were gradually coated by gold, the color of the solution changed the black nano-magnetite particles (A) to reddish brown (B) (Figure 4). The magnetic properties of the Fe_3_O_4_-gold nanoparticles can be controlled by synthesis conditions. For example, saturation magnetization values for uncoated and coated Fe_3_O_4 _nanoparticles can be decreased with the formation of gold layer at different temperatures [9].

**Figure 5:**
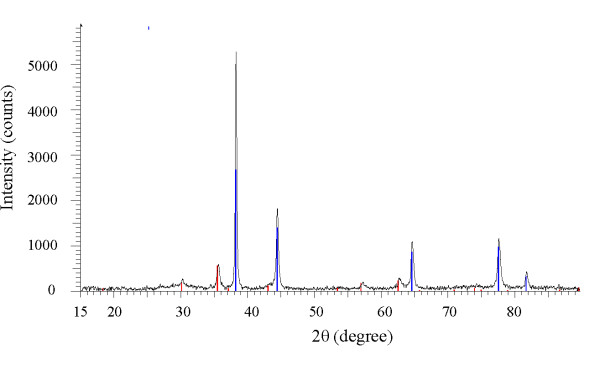
**XRD pattern of Core-shell Fe_3_O_4_-gold nanoparticles with 15 nm diameter**.

The XRD spectra of the Fe_3_O_4_-gold nanoparticles showed that they have an average diameter size of 15 nm. The diffraction peaks at 2*θ*° = 38.3°, 44.2°, 64.5°, 77.8°, and 81.7° are attributed to Fe-gold, which can be indexed to 111, 200, 220, 311, and 222 lattice planes of gold in a cubic phase, respectively. The absence of any diffraction peaks for Fe_3_O_4 _is most likely due to the heavy atom effect from gold as a result of the formation of gold-coated Fe_3_O_4 _nanoparticles. The diffraction peaks from Fe_3_O_4 _provide strong evidence for complete coverage of the magnetic core by gold (Figure 5).

### Physical properties of Fe_3_O_4_-gold-chitosan hydrogel nanocomposite

**Figure 6:**
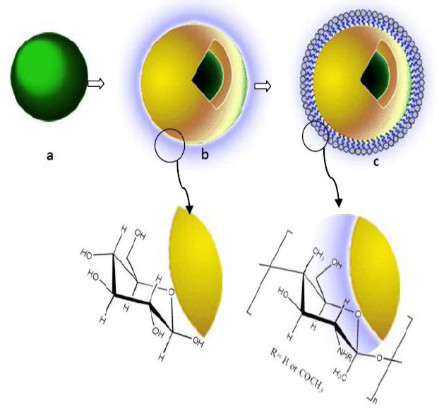
**The schematic of core-shell Fe_3_O_4_-gold-chitosan nanocomposite formation, a) magnetic Fe_3_O_4 _nanoparticles synthesized by co-precipitation method, b) gold shell propagated by electroless techniques with glucose as biological friendly reducing agent, and c) core-shell of Fe_3_O_4_-gold stabilized by chitosan with crowding method**.

**Figure 7:**
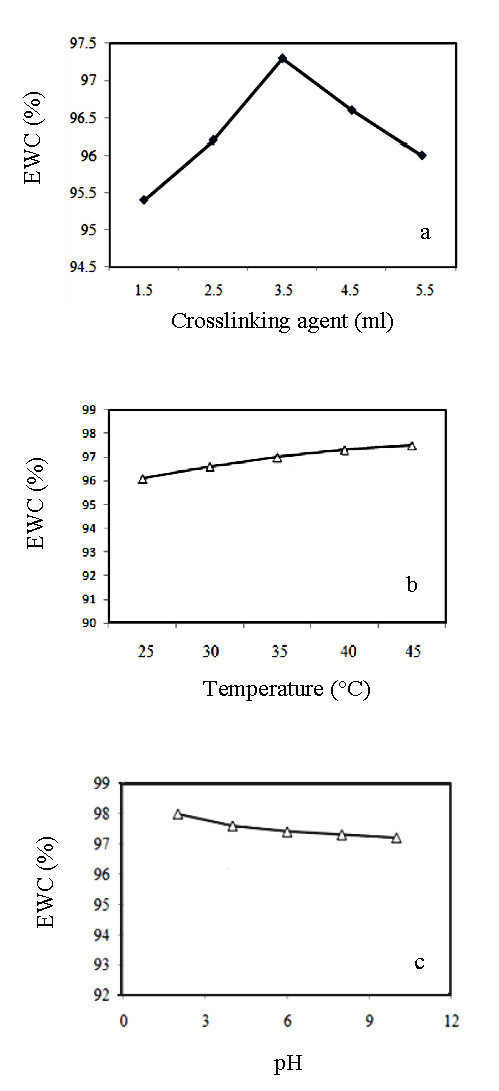
**Effect of various factors on the equilibrium water cntent of chitosan hydrogel**. a) different formaldehyde concentrations added at pH 7 and T = 35°C; b) pH = 7 and crosslinker = 3.5 ml formaldehyde 5 M; c) T = 35°C, crosslinker = 3.5 ml formaldehyde 5 M.

Chitosan plays an important role in nanocomposite production via amino and hydroxyl groups, and stabilizes the produced nanoparticles. It seems that Au^3+ ^ions were absorbed at first physically on the surface of Fe_3_O_4_, and then chemically by adding glucose and chitosan in order to retrieve its electron. Chitosan and glucose both act as reducing and stabilizing agents via the crowding method [16,17] (Figure 6). The effect of various parameters including the amount of formaldehyde as cross linker, pH and temperature on the equilibrium water content (EWC %) of the formed chitosan hydrogel was evaluated. When the concentration of formaldehyde was increased, the equilibrium water content decreased (Figure 7a). This can be due to a decrease in the space between polymer chains. The maximum EWC% of the hydrogel was observed at pH 3 (Figure 7c), this is attributed to complete protonation of the amine groups of chitosan. The hydrogel exhibited an equilibrium water content (EWC %) in the range of 96-97.5% at pH 7 and temperature between 25-45°C (Figure 7b). The chitosan hydrogel showed maximum swelling at low pH and high temperature.

**Figure 8:**
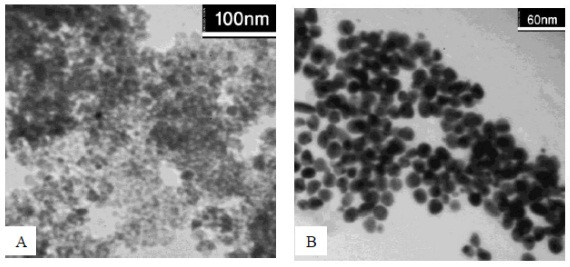
**TEM images of Fe_3_O_4 _nanoparticles (A), and core-shell Fe_3_O_4_-Au nanoparticles stabilized using chitosan (B)**.

**Figure 9:**
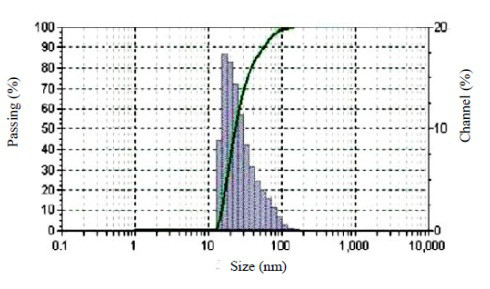
**The schematic of dynamic light scattering measurement for Fe_3_O_4_-Au nanoparticles stabilized using chitosan**.

The TEM image of the core shell Fe_3_O_4_-gold nanoparticles stabilized by chitosan confirms the formation of core-shell Fe_3_O_4_-gold nanoparticles (Figure 8). The Fe_3_O_4 _core, after it was coated with the gold shell, was much darker than the pre-coated magnetite nanoparticles. TEM analysis revealed that the average particle size increased from 9.8 nm before gold coating to 15 nm after gold coating, respectively. The average diameter of nanoparticles was found to be about 25 ± 5 nm using dynamic light scattering (DLS) measurements (Figure 9). Of course, it seems that DLS is not accurate method for true size measurement of nanoparticles. The synthesized nanoparticles were uniformly dispersed in the sample and seemed to be spherical in structure. To obtain a monotonous, smooth gold-layer shell, glucose was used to reduce Au^3+^. Ultrasonic agitation was applied to give it uniform monodispersity and to prevent particle aggregation.

Up until now, considerable effort has gone into the formation of gold-coated magnetite nanoparticles, but the use of them is still restricted due to some problems in the way it is synthesized [2,12,18,19]. In most cases, hydroxylamine, citrate, and borohydride have been used as reducing agents in combination with the reverse micelle technique for reducing gold salt nanoparticles [13,18,20,21]. Tamer et al. [19] reported a two-step synthetic method in which the magnetite nanoparticles were coated with gold using the borohydride reduction of HAuCl_4 _under sonication in order to achieve a better monodispersity and prevent aggregation problems. In this study, the use of the biopolymer chitosan as a template for the preparation of stable magnetite-gold core-shell monodisperse nanoparticles with a mean diameter of 15 nm was developed under mild temperature conditions.

## Conclusions

In summary, a magnetic core-shell-chitosan nanocomposite was synthesized. A rapid, simple, agglomerate-free method was reported for the production of monodisperse gold-coated Fe_3_O_4 _nanoparticles using biopolymer chitosan as a stabilizing agent. Core-shell magnetic Fe_3_O_4_-gold-chitosan nanostructures show a great potential for biotechnological and biomedical applications in the near future, especially for biodetection and bioimaging, drug delivery, and magnetic bioseparation.

## Competing interests

The authors declare that they have no competing interests.

## Authors' contributions

Professor HS was main supervisor of this research in University of Isfahan and wrote this manuscript when he was as visitor in University of Ottawa. MS was our MSc student and carried out many experiments. Dr. EH participated in experiments and effectively in writing paper. Professor KK from University of Ottawa contributed and supported in editing and completing this manuscript and gave us valuable guidance to improve this work. All authors read and approved the final manuscript.
